# Availability of Maternal, Newborn care and Child Health Services at Primary Health Care Unit during COVID-19 Outbreak in Ethiopia

**DOI:** 10.4314/ejhs.v33i2.5S

**Published:** 2023-10

**Authors:** Berhanu Fikadie Endehabtu, Dessie Abebaw Angaw, Tajebew Zayede Gonete, Meskerem Jisso, Netsanet Abera, Akalewold Alemayehu, Rekiku Fikre, Biru Abdissa, Abdurezak Umer, Mesfin Kebede, Hussen Mohammed, Bekele Yazie, Kassahun Dessie, Alemu Tamiso, Habtamu Sime, Elias Ali Yesuf, Kassu Ketema Gurmu, Binyam Tilahun

**Affiliations:** 1 University of Gonder, College of Medicine and Health Science, Institute of Public Health, Ethiopia; 2 eHealthLab Ethiopia, University of Gondar, Ethiopia; 3 Hawassa University, College of Medicine and Health Sciences, Ethiopia; 4 Jimma University, Institute of Health, Jimma, Ethiopia; 5 Dire Dawa University, College of Medicine and Health Sciences, Ethiopia; 6 World Health Organization Country Office for Ethiopia, Universal Health Coverage/Life Course, Health System Strengthening Team, Ethiopia

**Keywords:** Availability, Maternity, new born care, child health, region, essential health service, Ethiopia

## Abstract

**Background:**

The COVID-19 pandemic is putting a pressure on global health systems. The disruption of essential health services (EHS) has an impact on the health of mothers, neonate and children in developing countries. Therefore, the main aim of this study was assessing the availability of Maternal, Newborn care and Child health (MNCHS) services at primary health care unit during COVID-19 outbreak.

**Methods:**

A cross-sectional survey was conducted in five regions of Ethiopia in 2021. Descriptive analyses were undertaken using STATA 16 software and the results presented using tables and different graphs. A continuity of EHS assessment tool adopted from WHO was used for data collection.

**Result:**

During COVID -19 pandemic, 30 (69.8%) of woreda health offices, 52 (56.5%) of health centers (HCs), 7 (44.4%) of hospitals, and 165 (48%) of health posts (HPs) had a defined list of EHS. In comparison with other EHS, family planning is the least available service in all regions. At HPs level care for sick children and antenatal care (ANC) were available at 59.1 and 58.82% respectively. Except immunization services at SNNP, all other maternal, newborn, and child health EHS were not available to all HPs at full scale.

**Conclusion:**

Immunization services were most available, while ANC and care for sick children were least available during COVID-19 at the HPs level. There was regional variation in MNCH EHS service availability at all levels.

## Introduction

Health systems in low and middle-income countries, such as Ethiopia, may be vulnerable to the pandemic's caseloads and priority setting(1). It is crucial to provide key maternity, newborn, and child health (MNCH) services in order to ensure positive mother and child health outcomes.

Reproductive, Maternal, Newborn, and Child Health (RMNCH) refers to health challenges and interventions that affect women before, during, and after pregnancy, as well as newborns, infants, and children(2). The majority of maternal, neonatal and child deaths and morbidities are known to be avoidable with simple interventions. However, unavailability and low usage of essential health services continue to be substantial obstacles to the mothers' newborns' and child's optimal health. Despite the unreserved efforts of governments and other partners, the maternal mortality ratio and infant mortality rate in Ethiopia and other low-income countries remain unacceptably high(2-6).

Endorsed by the World Health Assembly in 2020, the Immunization Agenda 2030 (IA2030) strives to reduce morbidity and mortality from vaccine-preventable diseases across the life course(7). Vaccination has been one of the most effective interventions in driving down infant mortality to historically low levels worldwide(8).

Vaccines prevent an estimated 2.5 million deaths among children under five every year. Most unvaccinated children live in the poorest countries and are disproportionately in fragile or conflict-affected states. Almost half of them are in just 16 countries, including Ethiopia(7).

Despite increasing accessibility of services, uneven distribution of health resources, sub-optimal quality of care, low child health care seeking behaviors of communities, low coverage of kangaroo mother care (KMC) services, and shortage of essential health commodities and equipment at service delivery points remain to be key challenges contributing to high rates of neonatal and child mortality(4, 5).

Countries should establish the sort and mix of health services that will best meet the requirements of their citizens in order to proceed toward universal health coverage. In November 2019, Ethiopia launched the Essential Health Service Package (EHSP), which aims to ensure that all Ethiopians have access to high-quality health care regardless of their age, capacity to pay, economic status, or geographic location(9, 10). Interventions chosen to address the major causes of death, risk factors and diseases are detailed for key health services sub-components falling under each major component. Maintaining maternal, neonatal and child health is one component of countries EHS package(11, 12).

The coronavirus disease 2019 (COVID-19) pandemic is the worst global health crisis of a century declared as a Public Health Emergency of International Concern by the World Health Organization (WHO) in March 2020. Ethiopia reported the first confirmed COVID-19 case on 13th March 2020. The COVID-19 pandemic has challenged the resilience of the most effective health systems in the world. In many settings, health care service provision has been modified to focus on managing COVID-19 cases, and this pandemic focused approach has been affecting the provision of routine health services including RMNCH services (13, 14).

A well organized and prepared health system has the capacity to maintain equitable access to high-quality essential health services throughout an emergency, limiting direct mortality and avoiding indirect mortality and morbidity(10).

The COVID-19 pandemic threatens to disrupt the provision of essential services due to barriers to the supply and demand for services. As a result of disruptions in all essential services, child mortality in Ethiopia could increase by 15 percent and maternal mortality by 8 percent over the next year(6). Maintaining essential health services during the COVID-19 pandemic is critical to prevent these severe outcomes and protect the gains made over the past years in reducing maternal and child mortality(10). Therefore, the aims of this study was assess the availability of maternal, newborn care and child health services at primary health care units in Ethiopia during COVID-19.

## Materials and Methods

**Study design, setting, and period:** A national cross sectional survey was conducted to assess availability of maternal, newborn care and child health services at primary health care unit during COVID-19 outbreak in Ethiopia at four regions and one city administration namely: Namely Amhara, Southern Nation and Nationalities of People (SNNP), Sidama, Oromia region and Dire Dawa City Administration in 43 districts

The data were collected at four regions of Ethiopia and one city administration between November 8 and December 6, 2021.

**Study population, sample size and sampling procedures:** The study included health centers, hospitals, woreda health offices and health posts from the selected districts of the four regions and one city administration.

Woredas were selected for the assessment jointly with the regional health bureau and all primary hospitals were incorporated in the study from the selected districts whereas health centers and health posts were selected randomly by using lottery method Over all, a total of 92 health centers, 16 primary hospitals, and 344 health post were involved in the survey from the four regions and one city administration.

Essential health service availability as fully, partially, or no service during covid-19 pandemic were the major outcome variables. According to the Ethiopian essential health service guideline, the EHS is fully available, when all components of each service are available or not functional in the defined facility. Partially available or functioning essential health services, on the other hand, are when at least one component of a service is not available or not functional in the defined facility since the services are required; otherwise, not available or functional service. Under these, availability of family planning and contraception, antenatal care, labor delivery and newborn care, postnatal care, immunization services, care for sick children, and diagnosis and treatment of malnutrition are the outcome variables.

**Data collection tools, procedures, and data quality assurance:** A continuity of essential health services assessment tool adopted from WHO was used to assess availability of maternal, newborn and child health services at primary health care unit during COVID-19 outbreak in Ethiopia (15).

The assessment tool covers the following aspects of essential health services: Family planning and contraception, antenatal care, labor delivery and newborn care, postnatal care, Immunization services including vaccine readiness and availability, care for sick children, and diagnosis and treatment of malnutrition.

Fifty-four data collectors and 17 supervisors were trained for three days on the questionnaire using the electronic platform. Both electronic (Kobo collect) and paper-based data collection tool were administered through face-to-face interview. Interview with heads of the selected facilities and focal persons of each service unit were undertaken to collect data.

Pre-test was also undertaken prior to the actual data collection. The *Kobo Collect* form was prepared with strict rules to assure consistency of entry across the questions and legal value entries for responses. Close follow up by the research team and supervisors was undertaken. The completed forms were downloaded on daily bases to monitor the progress as well as the completeness of the form. Feedbacks were also given on the completed forms for the data collectors before the next data collection day.

**Data management and analysis:** The data were exported from the Kobo collect to Excel then to STATA. Descriptive analyses like frequency, percentage, and cross tabulations were undertaken and the results were presented using tables and different graphs to describe the services available and the gaps identified.

**Ethics approval and consent to participate data:** Ethical clearance was obtained from the Institutional Review Board of University of Gondar, Jimma University, Hawassa university and Dire-Dawa Universities. Official permission letter from the Regional Health Bureau was secured to Zonal health departments, Woreda Health offices and health facilities. Informed consent was obtained from all study respondents and participants after adequate information about the study had been provided. The collected data were treated and kept confidentially.

## Results

**Organization Characteristics:** The assessment was conducted at four regions and one city administrative namely: Amhara, Oromia, Sidama, South Nation and Nationalities of People (SNNP) region and Dire Dawa City Administration. The assessment was conducted at primary health care units, at 43 woreda health offices, 16 primary hospitals, 92 health centers, and 344 health posts (Table 1).

**Table 1:** Profile of assessed health facilities and organizations, Ethiopia, 2021

Region	#Health post	#Health Center	#Hospital	Total facilities assessed
Oromia	64	32	3	99
Amhara	66	10	7	83
SNNP	141	22	3	166
Sidama	40	13	3	56
Dire Dawa	33	15	0	48
**Total**	344	92	16	452

Fifty-four percent (54%) of health centers were urban, while 305 (88.7%) of health posts were rural. Only 42 (45.7%) of the 92 health centers provide in-patient services. There was no isolated labor and delivery room in nine (9.8%) of the health centers. Only seven (7.6%) of the health centers have an operating room, and all have a 24-hour staffed dedicated emergency unit (Table 2). Inpatient and operation services were more available in urban health centers than in rural health centers (Table 2).

**Table 2:** Organizational structure and health facility governance, Ethiopia, 2021

Characteristics	Number	%
Location of health centers		
Urban	50	54.3
Rural	42	45.7
Location of health post		
Urban	39	11.3
Rural	305	88.7
Health center governance		
Board	51	55.4
PHCU directors	41	44.6
Hospital Governance		
Board	15	93.8
Chief Clinical Officer (CCO)	1	6.2
Health center Services (n= 92)		
Inpatient service	42	45.7
Staffed emergency units	92	100
Operation unit	7	7.6
Isolated Labor and delivery room	83	90.2
Primary hospital (n= 16)		
Staffed emergency unit	16	100
Operation unit	14	87.5
Medical ward	16	100
Isolated labor and delivery	15	93.8
Surgical ward	16	10
NICU	16	100

NICU = Neonatal intensive care unit, PHCU=primary health care unit.

**Availability of essential health service list at PHCU:** During covid-19 pandemic, 165 (48%) health posts, 52 (56.5%) health centers. Thirty-five (81.4%) woreda health offices, 67(73%) the health center, 11(68.8%) hospitals, and 207 (60%) of health posts had a defined list of essential health services before the COVID -19 pandemic. Before the covid-19 pandemic 30 (69.8%) woreda health offices, 229 (66.5%) of health posts, and 58 (63%) of health centers have received a defined list of EHS.

### Availability of Maternal, New born care, and child health services

***Maternal, New born care, and child health services availability at primary hospitals:*** The assessment showed that delivery, family planning, immunization, diagnosis and treatment of malnutrition, and care for sick children were not delivered at full scale. Only 11 (68.8%) hospitals were providing full services for diagnosis and treatment of malnutrition, and delivery service (Table 3).

**Table 3:** Percentage of maternal, new born care and child health services deliver at hospitals, in Ethiopia, 2021 (n= 16)

Services	Fully (%)	Partially (%)	No (%)
ANC	100	0	0
Labor, delivery and new born care	81.3	18.7	0
Postnatal	94.4	5.6	0
Family planning	83.3	16.7	0
Immunization	89	5.5	5.5
Care for sick children	81.3	18.7	0
Diagnosis and treatment of malnutrition	81.3	6.2	12.5

***Availability of maternal, new born care and child health services at Health Centers:*** According to the findings of this study, most health centers are providing full-scale maternal, newborn, and child health services. Only 13 percent of health centers had fully available services for diagnosis and treatment of malnutrition, despite the fact that postnatal care and immunization were provided at full scale in 93 percent and 95 percent of health centers, respectively.

***Availability of maternal, new born and child health service with urban and rural setting at health centers:*** Labor and delivery, care for sick children, and immunization service are uniformly fully available at both facilities. Considering diagnosis and treatment of malnutrition at rural site the availability at full scale was 46.5% (Table 4).

**Table 4:** Availability of maternal, new born care and child health services by urban and rural health centers in Ethiopia, 2021

EHS	Urban (n= 49)			Rural (n= 43)		

	Available at full scale (%)	Available Partially (%)	Service is not available (%)	Available at full scale (%)	Available Partially (%)	Service is not available (%)
Family planning	87.8	12.2	0	72	28	0
Ante natal care	85.7	14.3	0	90.7	9.3	0
Labor delivery and new born care	81.6	18.4	0	81.4	14	4.6
Post-natal care	91.8	8.2	0	95.3	4.7	0
Immunization service	96	4	0	97.7	2.3	0
Care for sick children	89.8	10.2	0	90.7	9.3	0
Diagnosis and treatment of malnutrition	79.6	12.2	8.2	46.5	28	25.5

***Maternal, new born care and child health service availability by health posts:*** The study showed that, immunization, posts natal care and family planning services were available at full scale at 92.7%, 82.7% and 70.3% of health posts respectively. Whereas ANC and care for sick children were available at full scale 58.82% and 59.09% of health posts respectively, which was the least available EHS at health posts level compared with other EHS.

***Regional variation in maternal, new born care and child health service availability at health centers and health posts:*** Apart from immunization services, no other maternal, newborn, or child health services were available at full scale across the SNNP region's HPs. Health posts in the Dire Dawa, SNNP, and Sidama areas have a high availability of essential health services, but HPs in the Oromia and Amhara regions have a low availability of EHS. Except for sick children's care, all health centers under the Dire Dawa city government have provided complete coverage of all maternity, newborn, and child health vital health services. The availability of maternity, newborn, and child health essential health service in Amhara region health centers was poor. (Table 5).

**Table 5:** Availability of maternal, new born care and child health services at health posts and health centers across regions, Ethiopia, 2021

Service				Health Post (n= 344)											

	Oromia			Amhara			SNNP			Sidama			Dire Dawa		

	A	B	C	A	B	C	A	B	C	A	B	C	A	B	C
Family planning	54.7	43.8	1.5	34.8	57.6	7.6	49.6	32	18.4	75	22.5	2.5	85	12	3
Ante natal care	18.8	53.1	28.1	30.3	59.1	10.6	58	37	5	90	10	0	97	0	3
Post natal care	50	31.3	18.7	81.8	15.2	3	92.9	6.4	0.7	82.5	15	2.5	94	3	3
Immunization	76.6	14	9.4	89	4.5	7.5	100	0	0	97.5	2.5	0	97	3	0
Care for sick children	28.2	48.4	23.4	33.3	53.1	13.6	77.3	26.2	3.6	62.5	30	7.5	94	3	3
		**Health Center (n= 92)**													
Service	A	B	C	A	B	C	A	B	C	A	B	C	A	B	C
Family planning	75	25	0	60	40	0	86.4	13.6	0	69.2	30.8	0	100	0	0
Ante natal care	81.3	18.7	0	70	30	0	95.4	4.6	0	92.3	7.7	0	100	0	0
Labor, delivery and new born care	68.8	25	6.2	50	50	0	95.4	4.6	0	92.3	7.7	0	100	0	0
Post natal care	93.8	6.2	0	70	30	0	95.4	4.6	0	100	0	0	100	0	0
Immunization	100	0	0	70	30	0	90.8	4.6	4.6	100	0	0	100	0	0
Care for sick children	97	3	0	50	50	0	86.4	13.6	0	92.3	7.7	0	93.3	6.7	
Malnutrition diagnosis& treatment	40.6	28.1	31.3	70	10	20	69.2	15.4	15.4	81.8	4.6	13.6	93	7	0

Keys: A = Service is available at full scale, B = Service is available partially, C = Service is not available

***Regional variation in maternal, new born care and child health essential health service availability at primary hospitals:*** All maternal, new born care and child health essential health services were available at full scale at SNNP, Sidama region and Dire Dawa city administration hospitals and four EHS ( ANC, labor, PNC, care for sick children and malnutrition diagnosis and treatment) were also available at full scale at Oromia region primary hospitals. Whereas, except ante natal care service all other maternal new born and child health essential health services were not available full scale at Amhara region hospitals (Table 6).

**Table 6:** Availability of maternal, new born care and child health at primary hospitals across regions, Ethiopia, 2021

Service		Primary Hospital (n= 16)										

	Oromia			Amhara			SNNP			Sidama		
			
	A	B	C	A	B	C	A	B	C	A	B	C
Family planning	67	33	0	71.4	28.6	0	100	0	0	100	0	0
Ante natal care	100	0	0	100	0	0		0	0	100	0	0
Labor, delivery and new born care	67	33	0	57	43	0	100	0	0	100	0	0
Post natal care	100	0	0	85.7	14.3	0	100	0	0	100	0	0
Immunization	67	33	0	85.7	14.3	0	100	0	0	100	0	0
Care for sick children	100	0	0	71.4	28.6	0	100	0	0	100	0	0
Diagnosis and treatment of Malnutrition	100	0	0	57.1	14.3	28.6	100	0	0	100	0	0

Keys: A = Service is available at full scale, B = Service is available partially, C = Service is not available

***Availability of cold chain equipment in the selected Health Posts:*** The study showed that, 86 (51.19%) of those HPs with vaccine fridges have a temperature recorder/logger and from these only 49 (56.97%) of them have a functional temperature logger/recorder. Regarding the presence of a cold box; 199 (56.06%) of the HPs have a cold box, and of these 163 (81.91%) have a functional cold box. From those, 145 (72.86%) have 1 to 2 cold boxes. Moreover, the availability of ice packs for each of the cold boxes; 156 (78.39%) of the HPs have an ice pack and of these 120 (76.92%) of them have a set of ice packs for all cold boxes while the rest 43 (21.61%) of the HPs doesn't have a full set of ice packs for each of the cold boxes. Of the total 344 health post, 304 (88.89%) of them had at least one vaccine carrier in their facilities however 38 (11.11%) health post had no vaccine carrier in the facilities. Regarding the availability of safety boxes in the facilities, almost all health post (97%) had safety boxes.

***Availability of cold chain equipment in the selected Health Centers:*** The study showed that, all HCs had vaccine refrigerator, 91(97.83%) of HCs had a temperature recorder/logger and from these majority (93.33%) of them had a functional temperature logger/recorder. Regarding the presence of a cold box; all of the 92 HCs (100%) have a cold box, of these majority (97.83%) have a functional cold box. In line with this, only 36 (40%) of the HCs have 3 to 8 cold boxes. The study also revealed that, 89 (96.74%) of the HCs have an ice pack and of these the majority (92.13%) of them have a set of ice packs for all cold boxes while the rest 3 (3.26%) of the HCs doesn't have a full set of ice packs for each of the cold boxes.

From the total of health centers assessed on their vaccine readiness, almost all health centers 90 (98%) had at least one vaccine carrier in their facilities and only 2 health centers have no vaccine carriers in their facilities. Regarding set of ice packs for each of the vaccine carriers from 90 health centers that have vaccine carries 81(90%) health centers have a set of ice packs for their all vaccine carries in the facilities but only 10% of health centers a set of ice packs only for some carrier's vaccine carries which found in the facilities.

***Availability of cold chain equipment in the selected Primary Hospitals:*** Of the total of 16 primary hospitals (PHs) with vaccine fridges, 15 (93.75%) have temperature recorder/logger and from these majority (93.33%) of them have functional temperature logger/recorder. Regarding the presence of a cold box; 14 (87.5%) of the PHs have a cold box, and of these majority (92.86%) have a functional cold box. From those 13 PHs who have a functional cold box, only 4 (30.77%) of the PHs have 3 to 8 cold boxes. Additionally, availability of ice pack for each of the cold boxes; 9 (69.23%) of the PHs have an ice packs and of these majority (77.78%) of them have a set of ice packs for all cold boxes while, the rest 2 (22.22%) of the PHs doesn't have a full set of ice packs for each of the cold boxes.

Regarding vaccine carrier capacity of the 16 selected primary hospitals, 14 (87.5%) have vaccine carries. From this most of the hospital 11(78.57%) contain 1 to 5 vaccine carries in their facility. From 16 selected primary hospitals, 93.75 % hospitals have safety boxes on their facilities.

### Availability of vaccines in the selected PHCUs

All the observed PHCUs (452) were assessed on the availability of seven vaccine types. About 83(90.2%) of the HCs had Pentavalent vaccine and other types and only 44.8% of the HPs had Pentavalent vaccine but didn't have measles, OPV, BCG, PCV, Rota, and TT vaccines. From the total of 16 primary hospitals, 15 PHs reported having six vaccines except OPV.

## Discussion

Regardless of the COVID-19, maintaining the health of mothers, newborn care, and child health is an important aspect of the health sector. Our finding revealed that only 42 (46%) health center provides in-patient service. This finding is supported by a study conducted in Kenya during COVID-19 pandemic(16). The possible reason might be lack of infrastructure such as shortage of class room and beds. Additionally, likely due to changes in patient and provider behaviors, suspension of health facilities or their non-emergency services, massive mobility restrictions, and the potential reduction in the risk of non-SARS-COV-2 diseases(17).

According to the current assessment, the number of EHS received at all health facilities, including health posts, health centers, and primary hospitals was declining during COVID-19. This finding was also supported by a prior Ethiopian study. The reason may might be; when health services are overburdened by epidemics, distribution of amended or prior EHS guidelines may be reduced(18).

According to the current finding, only two-third of hospitals were providing full services for diagnosis and treatment of malnutrition, and delivery service. Lack of supplies and drugs, lack of rooms, beds, trained personnel and skill gaps might be the possible reasons for services not delivered at full scale

Every health center in Ethiopia could deliver on a full scale, according to the Ethiopian essential health service guideline. In 16 and 21% of health centers, labor, delivery, and new-born care were not provided at all. Malnutrition diagnosis and treatment were the least fully available service at a health center, accounting for 13%.

The common reasons for services not available at full scale at health centers might be stoke out of drugs and supplies, skill gap, fear of complications, no food service at HCs, shortage of rooms, limited laboratory services, shortage of man power, low rate of monitoring and evaluation and covid-19 prevention measures(19). Considering maternal, new born and child health service variation across urban and rural health centers; the current finding showed that a clear significant difference on the availability of full-scale service for Family planning and diagnosis and treatment of malnutrition. For instance, at urban health facilities the availability of Full scare service for diagnosis and treatment of malnutrition was about 80%, whereas, it was 46.5 at a rural health center. This significant difference might be due to skill gap and lack of trained staff, shortage of supplies and medications, shortage of rooms for SAM management at the rural health center. Considering family planning, the difference might be due to lack of equipment and trained staffs (Immediate postpartum family planning, long acting family planning: intrauterine device)-), shortage of supplies like FP methods (IUCD, injectable), low rate of monitoring and evaluation, and shortage of human resource and rooms.

In the current assessment, the study revealed that one third of the health posts were partially delivered for ANC and care for sick children during COVID-19. This finding is also consistence with a study conducted in North Shewa in which essential health services (ANC, care for sick children) were declined during pandemic diseases compared to before COVID-19(20). The possible reason might be Shortage of medical equipment's, supplies and drugs, skill gap, the health posts is located same site or with in HC, security problem (some health posts were robbed), lack of guideline, and COVID 19 pandemic are the commonly reflected reasons for the interruption of the services at health posts.

Considering availability of maternal, new born care and child health care at health posts across regions, we found that service delivery at full scale was very low in almost all services (Family planning, Ante natal care, post-natal care, Immunization, Care for sick children). The possible reasons might be lack of security which interrupts regular service, lack of medical equipment's ( thermometer, weight scale, BP apparatus, stethoscope),kill gap and shortage of contraception ( injectable, implants, condom, oral contraceptive(18, 21).

Regional variation for availability of EHS at full scale at primary hospital, the three regions namely: SNNP and Sidama had 100% performance. On the other hand, Amhara region had maximally 85% full scale service delivery except the antenatal care. It might be due to lack of trained staff and refrigerator, lack of medical equipment's, Shortage of medical equipment's (Delivery coach), political instability, and transportation problem (accessibility)(19, 22).

In this study, from the total facilities that have vaccine refrigerators, about 66.8% were functional refrigerators, and 31.9% of them had updated and functional temperature recording loger. WHO has recommended that the temperature range for vaccine storage should be 20 °C – 80 °C, to be read and recorded twice a day. This procedure helps for effective self-monitoring to prevent the breaking of the cold chain that can contribute to the primary of immunization services(6). Generally, 88.7% of surveyed PHCUs had vaccine carriers, of which only 10.4% reported having 6 and more carriers while 92.0% PHCUs had safety boxes. WHO recommended that countries must frequently reassess decisions to adapt immunization services and resume full services as soon as safely possible while monitoring vaccine stocks, related supplies, and catch-up needs (1).

All the observed PHCUs were assessed on the availability of seven vaccine types. 83(90.2%) of the HCs had the Pentavalent vaccine and other types and only 44.8% of the HPs had the pentavalent vaccine but didn't have Measles, Oral polio virus (OPV), BCG, PCV, Rota, and Tetanus toxoid (TT) vaccines. In 2019, the United Kingdom lost its measles “elimination status” because of outbreaks resulting from historically low vaccine uptake (6). Most unvaccinated children live in the poorest countries and are disproportionately in fragile or conflict-affected states. Almost half are in just 16 countries, Ethiopia is one (7).

Despite the fact that the current study included a large number of health facilities at the national level and used WHO tools with expertise modification as a strength, there was no evidence to show a temporal relationship between the level of essential health service status and factors that can act as enablers and barriers.

In conclusion, the availability of essential health services in PHCU has decreased during COVID-19 compared to before COVID-19. When compared to other services at the health center and health post level, the availability of immunization services available at full scale was highest among maternal, new born care, and child health services. In the current study, there was a clear full-scale essential health services availability variation across regions. ANC and care for sick children were the lowest full scale available service during COVID-19 at health post level. Additionally, there was a clear difference between urban and rural health centers on family planning and diagnosis and treatment of malnutrition at full scale service availability. Immunization service availability was relatively higher among PHCUs with vaccine storage capacity than that of their counterparts. It has also been shown that some facilities had equipment and vaccine shortages

The commonest reasons for not to have full scale service in all regions were lack of equipment and trained staffs, lack of laboratory diagnostics, shortage of rooms and of medications.

The major reasons for service unavailability were identified, so designing and implementing tailored intervention strategies or filling the gap in collaboration with stake holders would improve maternal, new born care and child primary health care units.

Strengthening primary health care linkage, availing, capacitating and motivating health work force, availing essential medicines and supplies would improve availability and service delivery capacity of health facilities, particularly at rural facilities family planning and diagnosis and treatment of malnutrition needs especial attention.
